# Effects of age-associated regional changes in aortic stiffness on human hemodynamics revealed by computational modeling

**DOI:** 10.1371/journal.pone.0173177

**Published:** 2017-03-02

**Authors:** Federica Cuomo, Sara Roccabianca, Desmond Dillon-Murphy, Nan Xiao, Jay D. Humphrey, C. Alberto Figueroa

**Affiliations:** 1 Department of Biomedical Engineering, University of Michigan, Ann Arbor, Michigan, United States of America; 2 Department of Mechanical Engineering, Michigan State University, East Lansing, Michigan, United States of America; 3 Department of Biomedical Engineering, King’s College London, London, United Kingdom; 4 Department of Biomedical Engineering, Yale University, New Haven, Connecticut, United States of America; 5 Vascular Biology and Therapeutics Program, Yale School of Medicine, New Haven, Connecticut, United States of America; 6 Department of Surgery, University of Michigan, Ann Arbor, Michigan, United States of America; University of Washington, UNITED STATES

## Abstract

Although considered by many as the gold standard clinical measure of arterial stiffness, carotid-to-femoral pulse wave velocity (*cf-PWV*) averages material and geometric properties over a large portion of the central arterial tree. Given that such properties may evolve differentially as a function of region in cases of hypertension and aging, among other conditions, there is a need to evaluate the potential utility of *cf-PWV* as an early diagnostic of progressive vascular stiffening. In this paper, we introduce a data-driven fluid-solid-interaction computational model of the human aorta to simulate effects of aging-related changes in regional wall properties (e.g., biaxial material stiffness and wall thickness) and conduit geometry (e.g., vessel caliber, length, and tortuosity) on several metrics of arterial stiffness, including distensibility, augmented pulse pressure, and cyclic changes in stored elastic energy. Using the best available biomechanical data, our results for *PWV* compare well to findings reported for large population studies while rendering a higher resolution description of evolving local and global metrics of aortic stiffening. Our results reveal similar spatio-temporal trends between stiffness and its surrogate metrics, except *PWV*, thus indicating a complex dependency of the latter on geometry. Lastly, our analysis highlights the importance of the tethering exerted by external tissues, which was iteratively estimated until hemodynamic simulations recovered typical values of tissue properties, pulse pressure, and *PWV* for each age group.

## Introduction

Since publication of the seminal study by Laurent et al. [1], it has become widely accepted that increased central artery stiffness is both an initiator and indicator of cardiovascular, neurovascular, and renovascular risk [2–4]. Among other effects, arterial stiffening increases the speed of propagation of the blood pressure wave along the central arteries (i.e., the pulse wave velocity, *PWV*), which results in an earlier return of reflected waves that augments blood pressure in the proximal aorta during systole (i.e., increases central pulse pressure, *cPP*). Increased *cPP*, in turn, increases the afterload on the heart during systole and decreases coronary perfusion during diastole. Although arterial stiffness is increased in multiple connective tissue disorders (e.g., Marfan syndrome [5,6]), diverse diseases (e.g., diabetes [7] and arthritis [8]), and hypertension [9], an otherwise inevitable cause of stiffening is normal arterial aging [10–12]. Common characteristics of large artery aging include endothelial dysfunction (i.e., reduced nitric oxide bioavailability), decreased smooth muscle cell function (including phenotypic modulation), damage to or degradation of elastic fibers, accumulation of glycosaminoglycans, and remodeling of fibrillar collagens [13–15]. Collectively, such changes in cellular function and extracellular matrix integrity affect local wall stiffness and manifest as altered hemodynamics.

The current gold standard clinical measure of arterial stiffening is the carotid-to-femoral pulse wave velocity (*cf-PWV* [16,17]), but other metrics (e.g., the augmentation index related to *cPP*) have found utility as well [18,19]. Most assessments of such metrics have been based on large clinical studies that seek to correlate disease presentation with changes in the metric of interest, which has yielded considerable insight. Nevertheless, the causes and consequences of increased central artery stiffness are many, and there is a pressing need for a more systematic and objective means to assess effects of local versus global changes in arterial mechanics on the overall hemodynamics. Fortunately, a number of studies report in vitro measurements of the biaxial mechanical properties of the aorta as a function of location and age [e.g.,^20^–^22^] and recent advances promise to enable characteristic properties to be inferred in vivo [23]. In this work, we use such data on aortic properties to inform a novel computational model of hemodynamic changes as a function of age. We submit that computational modeling is a powerful tool for investigating potential effects of regional differences in arterial geometry and wall properties on pulse wave propagation and other clinical metrics of arterial stiffening.

Specifically, we establish a baseline model of a young, healthy human aortic tree and then examine in silico the effects of age-associated changes in aortic wall geometry and wall properties on the overall hemodynamics. Based on a consistent comparison of data from many papers on aortic properties [24], we consider effects of regional changes in vessel caliber, length, and tortuosity (conduit characteristics) as well as wall thickness and biaxial material stiffness (wall characteristics) on the local and global hemodynamics. Using a subject-specific anatomical model to perform the fluid-solid interaction (FSI) simulations within the aorta allows us to prescribe realistic values of distal resistances to capture appropriate flow splits to the branches. We show that there are strong regional differences in all computed hemodynamics and that the distribution of *PWV* along the aorta, unlike other metrics of stiffening, does not always mirror the prescribed distribution of circumferential tissue stiffness. Furthermore, our analysis highlights the importance of external tissue support on the biomechanics of the aorta and its main branches. It is only through iterative adjustment of this external tissue support that we identified regionally-varying levels of transmural pressure that enabled a matching of all hemodynamic and biomechanical data on stiffness, pulse pressure, and pulse wave velocity for the three different age groups ranging from 30 to 75 years of age.

## Methods

Medical imaging now provides exquisite information on overall luminal geometries and it is thereby easy to capture subject-specific geometries at many ages of interest. There are also growing databases on changes in molecular and cellular mechanisms within the aorta with aging [14,15] which manifest as the observed changes in aortic properties [24], aortic length [25], and hemodynamics [11]. Yet, no current data set includes subject-specific longitudinal medical imaging and associated in vivo assessments of arterial wall properties, perivascular support, and regional hemodynamics. Hence, our strategy was, first, to define a subject-specific model for a young, healthy subject and, second, to age this model numerically by prescribing population-averaged geometries and material properties. In this way, we could study parametrically the effects of local and global changes in mechanics without adding inherent complexity due to biological variability in the aorta from subject to subject. Illustrative results are thus provided for three numerically aged vasculatures, one each for 40, 60, and 75-year-old (yo) subjects.

### Subject-specific baseline geometric model, pressures, and flow

**Fig 1:**
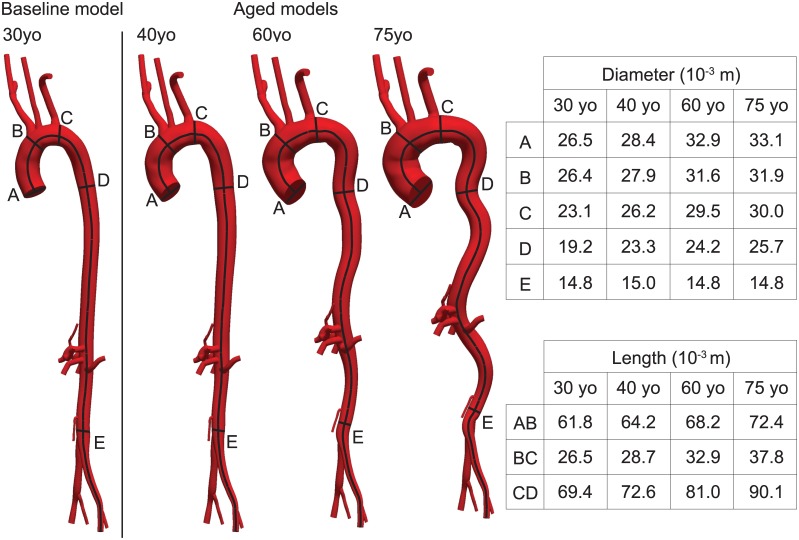
Schematics of the baseline 30 yo subject-specific model and the three numerically aged models corresponding to the 40, 60, and 75 yo groups. The tables summarize the prescribed values of regional inner diameters and aortic centerline lengths. All dimensions are given in mm.

Geometric data were extracted from magnetic resonance images collected previously for a healthy 30 yo male volunteer, with written consent from the individual and according to guidelines from the Institutional Review Board at King’s College London, UK. Briefly, the open-source modeling package CRIMSON (www.crimson.software) was used to construct the baseline geometric model, with centerline paths identified from the medical images along each vessel of interest and 2D contours drawn, with discrete spacing, perpendicular to each path to define the vessel lumen at those locations. Groups of contours were then lofted to create a 3D volume for each vessel (i.e., aorta and primary branches) and a union operation was used to merge the individual vessels into a single analytical geometric model [26]. See Fig 1 (left).

The mean arterial pressure (*MAP*) for this baseline subject was acquired noninvasively using applanation tonometry at the right carotid and right femoral arteries, also having obtained written consent from the individual and according to guidelines from the Institutional Review Board at King’s College London, UK. Central *MAP* was estimated from the right carotid pressure data whereas abdominal aortic *MAP* was estimated from the femoral artery data. Values for the baseline geometry at three select sites are listed in Table 1 along with values of blood pressure. Lastly, a flow waveform (mean 5.79 L/min, heart rate 60 bpm) was also collected non-invasively with phase contrast MRI under rest in the supine position for the same subject. We emphasize that, due to the lack of subject-specific regional material properties, we did not perform FSI simulations for this young subject; we merely, used these data to generate a baseline model that could be numerically aged. Clearly, there is a pressing need for reliable regional biaxial data for the aorta from young healthy subjects as well [24].

**Table 1:** 

Age (yo)	Location	Inner Radius (10^−3^ m)	Wall Thickness (10^−3^ m)	*MAP* (mmHg)	*EP* (mmHg)	In vivo Axial Stretch	In vivo Circ. Stretch	*K*_*θθθθ*_ (10^6^ Pa)	*K*_*ΖΖΖΖ*_ (10^6^ Pa)
30	ATA	13.2	1.80	89	-	-	-	-	-
	DTA	9.60	1.76	89	-	-	-	-	-
	IAA	7.81	1.14	91	-	-	-	-	-
40	ATA	14.07	2.00	93	7	1.15	1.34	0.40	0.28
	DTA	11.63	1.70	93	2	1.17	1.24	0.50	0.46
	IAA	8.24	1.05	95	0	1.23	1.24	1.46	1.30
60	ATA	16.13	2.45	95	17	1.08	1.22	0.75	0.27
	DTA	12.08	1.90	95	12	1.09	1.21	0.71	0.64
	IAA	8.27	1.50	97	0	1.09	1.12	1.66	1.51
75	ATA	16.25	2.90	102	15	1.03	1.14	0.91	0.41
	DTA	12.71	2.15	102	10	1.03	1.22	1.02	0.58
	IAA	8.98	1.92	105	0	1.04	1.01	2.14	1.97

Geometric parameters (inner radius) and wall properties (material stiffness and wall thickness) calculated at a transmural pressure given by the difference between mean arterial pressure (*MAP*) and external pressure (*EP*). Also listed are the in vivo axial and circumferential stretches as a function of three ages and locations, namely, the ascending thoracic aorta (ATA), proximal descending thoracic aorta (DTA), and infrarenal abdominal aorta (IAA). Material stiffness *K* is given in circumferential (θ) and axial (*Ζ*) directions.

### Age related changes in geometry, hemodynamics, and wall properties

There is considerable population-averaged information available on aging-associated changes in aortic geometry (namely, luminal diameter, segment length, and local curvature), blood pressures and flows, and wall properties (material stiffness and thickness of the wall). Whereas we merely extracted and used geometric and hemodynamic information directly from key papers [16,24,27,28], information on changes in regional mechanical properties required further analysis. Note, therefore, that we had previously recreated biaxial data from 6 published studies and performed a consistent nonlinear parameter estimation for a “four-fiber family” strain energy function that captures the biaxial stress-stretch behaviors [24]. Specifically, this phenomenological constitutive relation adopts a mass-averaged constrained mixture approach and defines strain-energy functions for individual structurally significant constituents: a neo-Hookean relation for the elastin-dominated amorphous matrix and a Fung-Type exponential for the collagen fiber-dominated anisotropy. For purposes of FSI simulations, a vessel of interest can then be modeled as a single-layered, thin-walled cylindrical tube, elongated to in vivo values of axial stretch and pressurized to transmural pressure (*TP*). That is, although the thin-walled assumption is not appropriate for studies of arterial wall mechanobiology [29], it yet provides the correct values of the mean wall stress (which reflect actual stress distributions since residual stresses homogenize the transmural distribution) and, importantly, the correct values of structural stiffness that are essential in FSI simulations. Toward that end, we used the theory of small deformations superimposed on large to compute in vivo relevant values of biaxial material stiffness [30], which when combined with wall thickness yields the structural stiffness in a computationally efficient manner [31]. By using this approach, we were able to refer the nonlinear constitutive behavior to an in vivo configuration, near the mean arterial pressure (MAP = P_dias_ + (P_sys_−P_dias_)/3), rather than needing to prescribe a stress-free configuration (often characterized by an opening angle that differs regionally and evolves with disease and aging).

Because best-fit values of the material parameters in this four-fiber family model were previously presented for three broad age groups (< 30, 31 to 60, and > 61), we re-evaluated the data to identify values for narrower age groups (30–49, 50–69, and 70–80 yo) and three primary regions of interest: ascending thoracic aorta (ATA), proximal descending thoracic aorta (DTA), and infrarenal abdominal aorta (IAA). The three aged models considered in this study (40, 60, and 75 yo) thus correspond to the mean ages of these three groups. Regional values of biaxial material stiffness over the physiologic range of age-specific diastolic-to-systolic pressures were then computed using the theory of small on large at the mean transmural pressure (*TP*) (i.e., the difference between *MAP* and external pressure (*EP*) due to perivascular tissue support) at age-specific values of in vivo axial stretch. Values of the computed biaxial material stiffness are listed in Table 1 for the three primary sites available. Table 2 further shows the published studies used herein to fit the model at each aortic location and age group. These studies are the same as those used previously [24], with the exception that data from [21] were not used to inform the model at DTA or IAA locations for the younger age groups since the resulting values of stiffness were much higher than those inferred from other studies.

**Table 2:** 

Age (yo)	Location	[21]	[22]	[32]	[33]	[20]	[34]
40	ATA	x		x			
	DTA					x	
	IAA				x		
60	ATA	x	x	x			
	DTA		x			x	
	IAA		x		x		
75	ATA	x	x				x
	DTA		x				
	IAA	x			x		

List of published studies in the literature used herein to define the model at each specific location and age group.

A multistep approach was then used to numerically age the subject-specific 30 yo model in a controlled manner: First, we increased the length of the aorta to reflect changes observed with aging. Craiem et al. [27] noted that this lengthening is not uniform and that, per each 10 years of aging, the ascending aorta (from the branching point of the left coronary artery to that of the brachiocephalic artery) exhibits a 3% increase in length, the aortic arch (from the brachiocephalic artery to the left subclavian) a 7% increase in length, and the descending aorta (from the left subclavian artery to the intersection of the thoracic aorta with a horizontal plane through the coronary sinus) a 5% increase in length (Fig 1). Using a custom algorithm in MATLAB (Mathworks, Natick, MA), the centroid for each aortic contour was calculated throughout the thoracic region and a 3D cubic spline was used to interpolate the centroids. This spline was then modified to reflect the changes in length with aging from 30 to 40, 60, and then 75 yo. Moreover, each aortic contour was shifted and rotated to lie at the same relative position to the new spline. Finally, for the branching vessels, the centroid of the contour nearest to the aorta was rotated and shifted to maintain the same position and orientation relative to the modified spline.

Second, we incorporated the expected slight decrease in subject height due to aging [28]. Using a method similar to that used to modify aortic length, we reduced the overall distance between the aortic root and the iliac bifurcation without altering the arc length of the aorta. This resulted in an increase in aortic tortuosity. Third, we incorporated directly reported regional changes in aortic diameter with aging [24]. The diameters of the subject-specific 30 yo model were increased with aging, scaling each contour relative to its position along the length of the aorta. Branching vessel diameters were uniformly scaled based on the scaling factor at the closest point on the aortic arc length (Fig 1). Fourth, starting from values of wall thickness for a 30 yo patient reported in [24], values of wall thickness in aging were chosen such that the same trend of inner radius to wall thickness ratio previously defined was maintained. Fifth, results for *MAP* for the 30 yo baseline subject (Table 1) were extrapolated for the older age groups following the previously found trend of pressure as a function of age (Fig 5 in [24]). Because there are no reported values of regional variations in the external pressure *EP* exerted on the vessel wall by the perivascular tissue, these values were estimated separately for each age group via an iterative approach detailed below in the “external tissue support” section.

### Computational framework

#### Numerical methods

Using an approach similar to that described previously [35,36], we employed a coupled-momentum method to model interactions between the arterial wall and blood flow [37]. This method is fundamental to model and capture arterial wave propagation and pulse pressure, but adds minimal computational cost to that of rigid wall formulations. Each arterial segment was thus modeled as an incompressible elastic membrane endowed with a thickness (to provide appropriate structural stiffness) and characterized by the appropriately linearized (over a cardiac cycle) biaxial tissue properties listed in Table 1. This linearization is justified by experimental evidence showing a near linear response over physiological ranges of pressure, particularly with age-associated stiffening, and is sufficient to compute values of PWV, one of the key goals of this paper [35,38–40].

#### Specification of vessel wall properties

**Fig 2:**
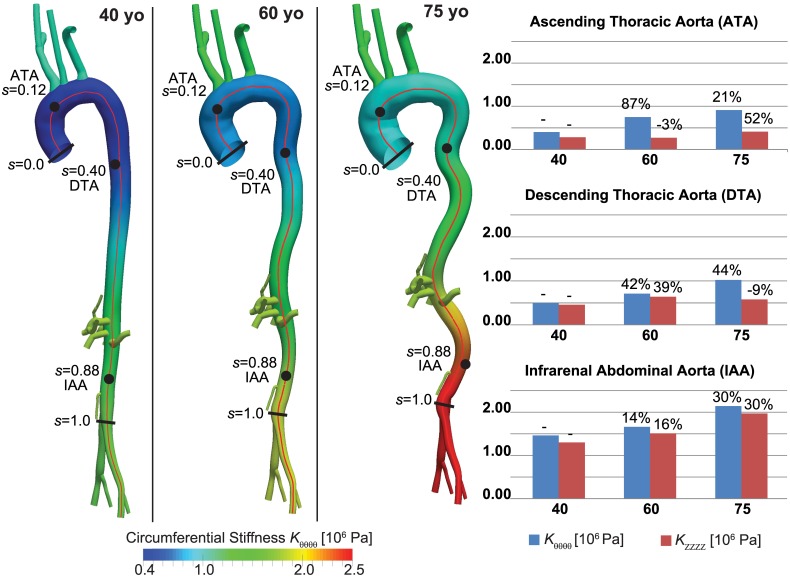
3D geometrical models for 40, 60, and 75 yo human subjects (*left*) and associated values of segmental biaxial material stiffness (MPa) in three regions (*right*): Ascending Thoracic Aorta (ATA), proximal Descending Thoracic Aorta (DTA), and Infrarenal Abdominal Aorta (IAA). Lengths along the aortic centerline are parameterized by *s* ∈[0,1], where *s* = 0 corresponds to the aortic root at the beginning of the ATA and *s* = 1 to the aorto-iliac bifurcation (left). Listed values of aortic material stiffness (right) were assigned at locations indicated by the black dots within the ATA, DTA and IAA and linearly interpolated to obtain a continuous distribution of stiffness, as illustrated colorimetrically on the aortic geometries for the circumferential values. Stiffness was assigned separately in circumferential (blue) and axial (red) directions at each location and for each age group. The % numbers refer to the percent difference in value relative to the prior age group.

The centerline path of the aorta was defined in terms of normalized arc-length *s* ∈[0,1] from the aortic root to the aorto-iliac bifurcation. Local values of biaxial stiffness and thickness were assigned at *s* = 0.12 for the ATA, *s* = 0.4 for the DTA and *s* = 0.88 for the IAA and interpolated linearly along the aorta (Fig 2). The values of biaxial stiffness and thickness were also extrapolated constantly from *s* = 0.12 to the aortic root and from *s* = 0.88 to the left and right iliac arteries. Stiffness values for the carotid artery were prescribed as a function of aging based on limited data in the literature [41], namely values of an “elastic modulus”. Given the lack of data, the subclavian arteries were assigned the same value of stiffness as the carotid arteries since they have comparable radii. Thus, the carotid and subclavian arteries are the only vessels in the model where the assigned tissue properties are isotropic. Note, therefore, that fully biaxial and anisotropic regionally-specific values were prescribed within the region of primary interest for the hemodynamics (i.e., the aorta) and that prescribing reduced-order 0D models (e.g., Windkessels that account for peripheral compliance) for all supra-aortic vessels minimizes the need to prescribe the 3D elastic properties of these vessels precisely.

In vivo measures of distensibility and *PWV* suggest that elastic arteries stiffen with aging whereas muscular arteries do not [42]. For this reason, we kept the stiffness of mesenteric, celiac, and renal arteries unchanged for the three age groups. Given the lack of any data to the contrary, we assumed that muscular aortic branches are stiffer than the aorta at a young age, but become comparable in stiffness when the aorta stiffens with aging [42]. Thus, these vessels were assigned a stiffness similar to that of the 75 yo abdominal aorta at the renal level for all age groups.

#### External tissue support

**Fig 3:**
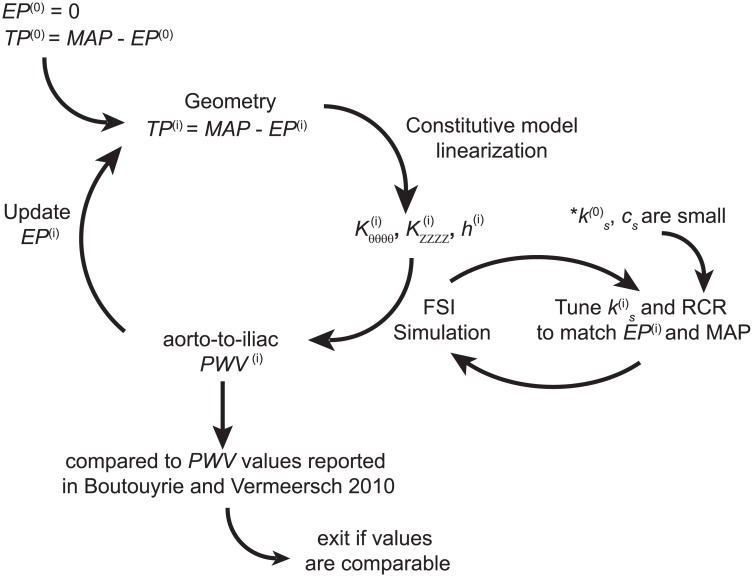
Iterative approach for tuning external tissue support parameters: Stiffness coefficient *k*_*s*_ and damping coefficient *c*_*s*_.

**Fig 4:**
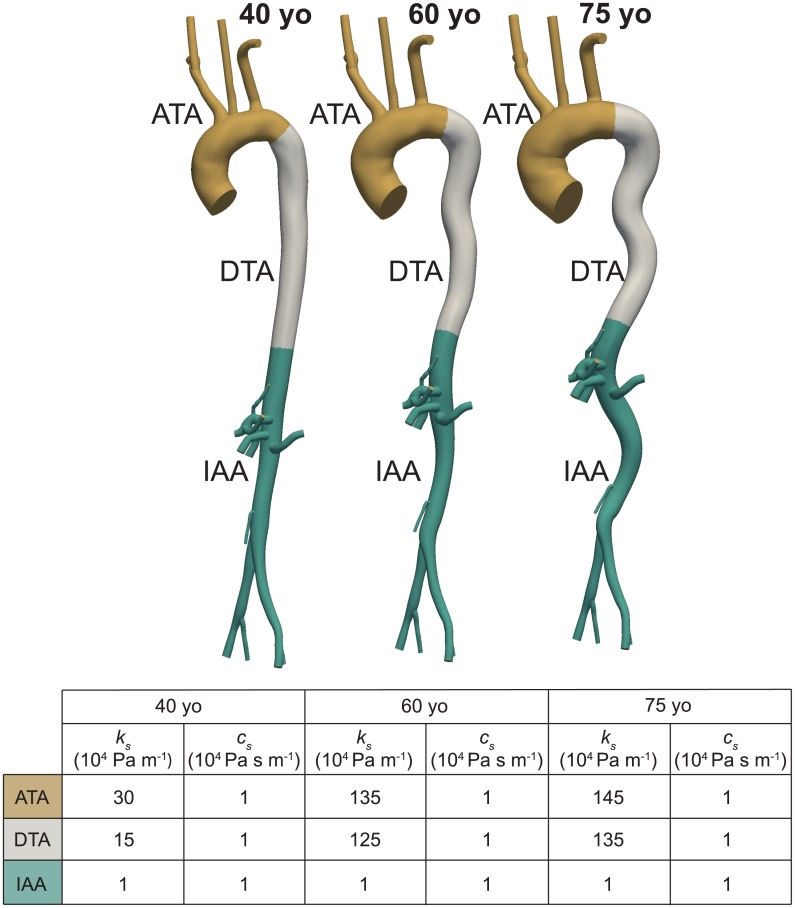
Spatial and temporal distribution of external tissue support parameters: Stiffness coefficient *k*_*s*_ and damping coefficient *c*_*s*_.

Arteries are supported by external tissues or organs, which constrain the motion of the vessels via an effective external pressure (*EP*). A simple traction boundary condition was applied on the outer side of the vessel wall to mimic the effects of a distributed viscoelastic force imparted by the perivascular tissue [43]. This traction boundary condition consists of a viscoelastic term with two distributed parameters: a stiffness coefficient (*k*_*s*_) and a damping coefficient (*c*_*s*_). From a computational perspective, this boundary condition helps to prevent high frequency oscillations (in contrast to pulsatile motions) of the wall, which arise in slender vessels under pulsatile flow and no external support [35]. The functional form of this traction is:

$$\sigma \;\cdot \;n=-k_{s}u-c_{s}v$$

where ***u*** and ***v*** are the displacement and velocity field, respectively. The weak form of the FSI problem including this boundary condition is given in [36]. Here, we adopted spatially variable values of external tissue support parameters and allowed the parameter values to change with aging. Due to the lack of experimental data on these parameters, an iterative approach was used to choose the values *k*_*s*_ and therefore the *EP* acting on vessel wall (Fig 3). The value of *c*_*s*_ was set to the minimum value that eliminates spurious oscillations in the arterial tree and was kept constant through the iterative process (see Fig 4).

Given the geometry and an initial guess of the *TP*^(0)^, the constitutive model was computed as described above. Initially, *EP* is set to 0 mmHg, therefore *TP*^(0)^ = *MAP*. An initial FSI simulation was then run with small values of *k*_*s*_ and *c*_*s*_ that produce an *EP* of approximately 0 mmHg. The computed aorto-iliac *PWV* was then compared to the values reported in [16]. If the computed aorto-iliac *PWV* values were too high, a higher value of *EP* was considered. This effectively reduced the *TP* and resulted in a lower local wall stiffness, especially in the circumferential direction. Given a new set of values for biaxial stiffness and thickness, the external tissue parameter *k*_*s*_ was iteratively estimated via subsequent FSI simulations until the new value of *EP* was matched. This iterative process was continued until the computed values of aorto-iliac *PVW* matched the values in [16] for each age group. During this iterative process we aimed to preserve our previous findings of increasing stiffness along the aorta for each age group [24]. To satisfy this stiffening pattern along the aortic length, while reproducing the reported values of *PWV* for each age group, a heterogeneous distribution of external tissue was needed, with the largest values in the thoracic region and smallest (zero) in the abdominal region for each age group. The final spatially-varying values of *EP* and external tissue support *k*_*s*_ are reported in Table 1 and Fig 4, respectively. For the purposes of external tissue support, the aorta was divided into three segments: ATA from *s* = 0 to *s* = 0.25, DTA from *s* = 0.25 to *s* = 0.66, and IAA for *s* > 0.66. The upper branches and lower branches received the same external tissue support as the ATA and IAA, respectively.

#### Inlet and outlet boundary conditions

The flow waveform (mean 5.79 L/min, heart rate 60 bpm) measured with phase contrast MRI in the 30 yo volunteer under supine, rest conditions was used as an inlet boundary condition. Since cardiac output remains nearly constant at resting conditions with aging [44,45], the same inlet flow was prescribed for all three aged models. Finally, outlet boundary conditions were prescribed using a coupled multi-domain method that represents the distal vasculature to the level of capillaries via individual three element (Resistance-Capacitance-Resistance) Windkessel models coupled to each outlet of the 3D models [46]. The parameters associated with these outlet conditions were tuned individually for each age group (Table 3) to ensure appropriate flow splits and to match the *MAP* used to calculate the linearized stiffness listed in Table 1 [35].

**Table 3:** 

	*R*_*prox*_			*R*_*dist*_			*C*		
	10^9^ Kg/(m^4^·s)			10^9^ Kg/(m^4^·s)			10^−9^ (m^4^·s)/Kg		
Vessel	40	60	75	40	60	75	40	60	75
	yo	yo	yo	yo	yo	yo	yo	yo	yo
Right Subclavian	0.2			1.60	1.66	1.75	1.68		
Left Subclavian	0.18			1.53	1.58	1.68	1.75		
Right Carotid	0.23			1.91	1.98	2.09	1.40		
Left Carotid	0.22			1.80	1.86	1.97	1.49		
Right Renal	0.24			1.12	1.16	1.24	2.43		
Left Renal	0.25			1.15	1.20	1.27	2.36		
Celiac Artery	0.35			1.53	1.59	1.70	1.78		
Gastric Artery	1.20			5.59	5.81	6.18	0.49		
Splenic Artery	0.55			2.50	2.60	2.76	1.09		
Superior Mesenteric	0.23			0.60	0.63	0.67	4.65		
Inferior Mesenteric	1.40			3.82	3.99	4.28	0.73		
Right Ext. Iliac	0.08			1.49			2.15		
Right Int. Iliac	0.29			5.50			0.56		
Left Ext. Iliac	0.08			1.49			2.15		
Left Int. iliac	0.30			5.45			0.56		

Outflow boundary conditions. Numerical values for the three-element Windkessel models prescribed at each branch. Values were adjusted following the procedure described in [35], and consist of proximal resistance (*R*_*prox*_), distal resistance (*R*_*dist*_) and compliance (*C*). *R*_*dist*_ was the only parameter that increased with aging. This increment was required to reproduce the reported increased values of *MAP* with aging.

## Results

**Fig 5:**
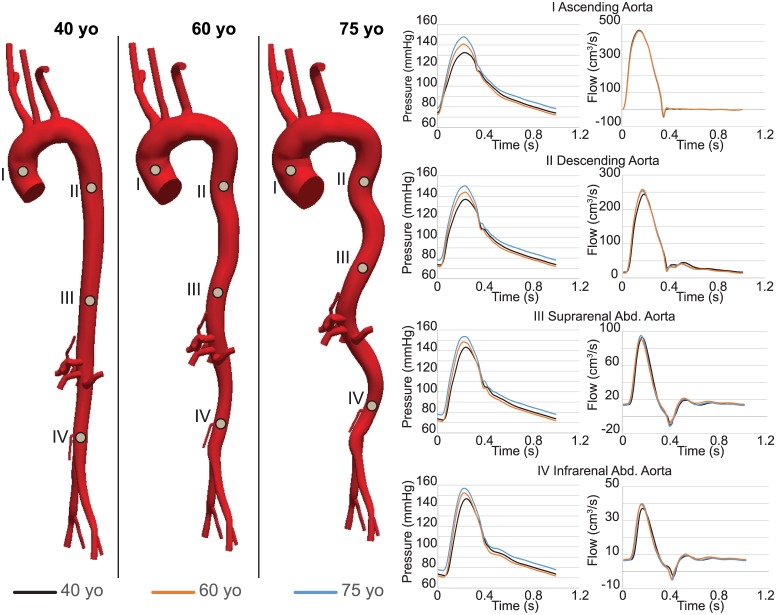
Pressure and flow waveforms for 40 (black), 60 (orange) and 75 (blue) yo subjects at four different locations along the aorta; namely ATA (I), DTA (II), SAA (III) and IAA (IV).

Fig 5 depicts pressure and flow waveforms at four different locations down the aorta for the different ages. Pressure waveforms show larger central pulse pressure and smaller pressure amplification for the 60 and 75 yo cases. Changes in flow waveforms are subtle. A good matching was obtained between the computed *MAP* and *EP* acting on the vessel wall and the *MAP* and *EP* used to calculate the linearized stiffness of the wall. The computed values of pressure are listed in Table 4. The computed *MAP* resulted mainly from tuning the distal resistance of the Windkessel parameters for each age group; this parameters increased the total resistance of the distal vasculature by 2.55% for the transition from 40 to 60 yo, and of 4.06% for the transition from 60 to 75 yo.

**Table 4:** 

Age (yo)	Location	*MAP* (mmHg)	*EP* (mmHg)
40	ATA	93.6	7.16
	DTA	94.6	1.76
	IAA	96.3	0.04
60	ATA	95.1	16.6
	DTA	95.9	10.4
	IAA	97.0	0.02
75	ATA	101.6	16.1
	DTA	102.2	8.9
	IAA	102.9	0.02

Computed mean arterial pressure (*MAP*) and external pressure (*EP*) for the three age groups at three locations: ascending thoracic aorta (ATA), descending thoracic aorta (DTA) and infrarenal abdominal aorta (IAA). The computed values match well the corresponding values in Table 1 used to calculate the linearized stiffness.

**Fig 6:**
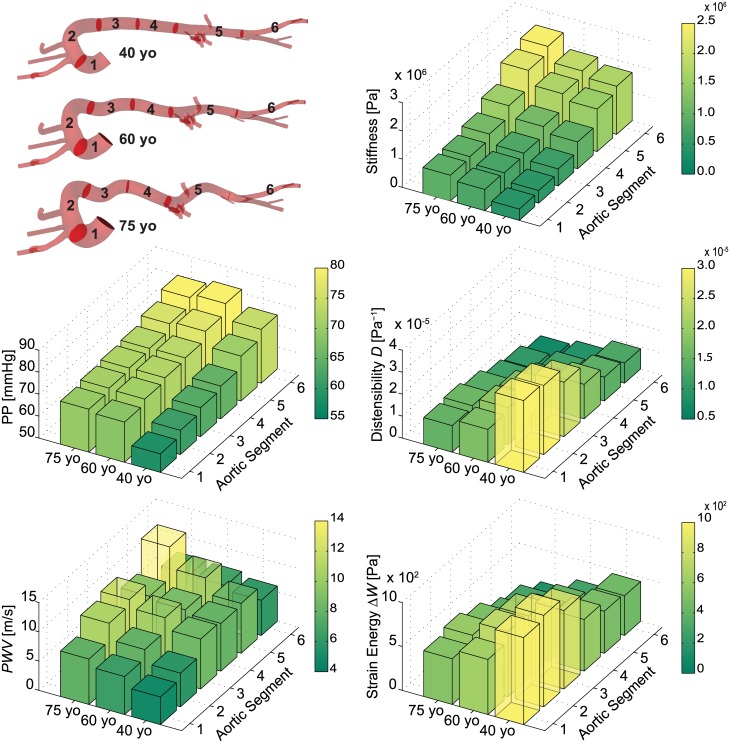
Computed values of circumferential material stiffness (Pa), local Pulse Pressure (*PP*, in mmHg), distensibility (Pa^-1^), Pulse Wave Velocity (*PWV*, in m/s), and change in strain energy storage (*ΔW*, in Pa x 100) as a function of region (aortic segments 1 to 6) for three different ages: 40, 60, and 75 yo. The 18 different computed values for each of the 5 metrics consistently show strong regional variations at each age.

Fig 6 compares the spatial distribution of four different metrics or consequence of arterial stiffening–local pulse pressure (*PP*), distensibility, pulse wave velocity (*PWV*), and changes in strain energy stored during the cardiac cycle (*ΔW*)–to the distribution of prescribed values of circumferential material stiffness. The first three of these four metrics are routinely measured clinically; the fourth of these metrics, stored energy, is motivated by recent findings in a mouse model of pre-mature aging in which changes in stiffness are due to a genetically defined connective tissue disorder [47]. Results are also shown for six different anatomical segments along the aorta: ATA from *s* = 0 to 0.12 (segment 1), aortic arch from *s* = 0.12 to 0.38 (segment 2), DTA from *s* = 0.38 to 0.55 (segment 3), suprarenal abdominal aorta from *s* = 0.55 to 0.71 (segment 4), IAA from *s* = 0.71 to 1 (segment 5), and iliac artery from *s* = 1 to 1.27 (segment 6). This visualization enables a spatially detailed description of the different metrics.

Distensibility was calculated using cross-section area, *D* = (*d*^*2*^_*sys*_*−d*^*2*^_*dia*_)/(*d*^*2*^_*dia*_*·PP*), rather than diameter, as is common in magnetic resonance imaging studies. The pulse wave velocity *PWV* was calculated as the ratio of the distance between two locations of interest and the transient time of the pressure pulse between these locations. In contrast to most clinical assessments, this distance was computed herein from the actual centerline of the vessel, not the straight line distance. The transient time between two waveforms was measured using an intersecting tangent algorithm that defines the foot of a pressure waveform as the intersection between the horizontal tangent intersecting its diastolic minimum and the tangent to the maximum systolic gradient; we used the algorithm developed in MATLAB by Gaddum et al. [48]. Finally, the change in strain energy throughout the cardiac cycle was calculated via the work done on the aorta given the assumption of no dissipation and no change in length: *ΔW* = 0.5(*P*_*sys*_*-P*_*dia*_)·(*A*_*sys*_*-A*_*dia*_)/*A*_*dia*_. The latter is an excellent assumption for all regions except the ATA, which experiences lengthening and shortening over the cardiac cycle, though data are lacking to quantify this as a function of aging.

Overall, the spatial distributions of our simulated values compared well with reported data on pulse pressure [49] and distensibility [24,50]. Local pulse pressure *PP* increased along the aorta: from 58 at the ascending aorta to 75 mmHg at iliac bifurcation in the 40 yo model; from 68 to 81 mmHg in the 60 yo model; and from 69 to 80 mmHg in the 75 yo model. These results reveal important features. As reported previously [49], there is an amplification of pulse pressure along the aorta, particularly at younger ages: a 17 mmHg pulse amplification at age 40 reduces to 11 mmHg at age 75. The pressure waveforms along the aorta thus become more alike in amplitude and contour with aging, which is to say that distal amplification attenuates with aging. Finally, we also reproduced the reported increase in *MAP* in the ATA with aging. This change resulted largely from the increased resistance in the 3D geometrical model arising from the increased tortuosity (cf. Fig 1) and the increased total peripheral resistance represented by the Windkessel models (cf. Table 3). Consistent with the literature, the largest predicted change in *PP* is between 40 yo and 60 yo, while 60 yo and 75 yo have more similar values. Indeed, it is at 50–55 yo that there tends to be, on average, dramatic changes in central pulse pressure and *cf-PWV* [10]. Overall, the computed regional patterns of *PP* follow the prescribed distribution of circumferential stiffness down the aorta for the three different ages. As expected, distensibility *D* decreases distally along the aorta at a given age, particularly in the younger healthy model. It similarly decreases with aging at each individual location, especially in the most proximal segments.

Unlike for the other three metrics of interest, the distribution of *PWV* along the aorta does not follow the prescribed distribution of circumferential stiffness everywhere (Fig 6). This difference arose, in part, because *PWV* results from both arterial geometry and stiffness. That is, *PWV* is influenced strongly by arterial tapering (which is not normalized as in the calculation of distensibility) and branching, and thus is difficult to intuit even with the aid of simplified equations such as Moens-Korteweg (which assumes uniform geometry and properties). Lastly, the distribution of changes in stored strain energy *ΔW* is comparable to the distensibility patterns.

Notwithstanding the utility of visual comparisons of spatio-temporal differences in the four clinical metrics of interest (Fig 6), we computed the correlation coefficient of each of these metrics with the prescribed circumferential material stiffness; this was done both spatially at a given age (Table 5) and temporally at a given location (Table 6). Briefly, using the software R (www.r-project.org/), Pearson correlation coefficients were computed as the ratio of the covariance to the product of the standard deviations of the two variables; hence a value of 1.0 reveals a perfect correlation, with positive and negative values indicating proportional and inverse correlations, respectively. At any given age, the spatial correlation with circumferential material stiffness is greater than 0.9 for *PP*, *D*, and *ΔW*, but not *PWV*, which has correlation values smaller than 0.3. At any given location, the correlation with circumferential stiffness is greater than 0.9 for all metrics with the exception for *PP*, where the correlation decreases to 0.7 for segments 3 and 4 and to 0.5 for segments 5 and 6. This finding reflects the relatively constant values of *PP* in the distal segments of the aorta with aging, despite the increase in tissue stiffness. A visual examination of the rows and columns of each bar plot in Fig 6 confirms the correlation coefficients reported in Tables 5 and 6.

**Table 5:** 

	40 yo	60 yo	75 yo
***PP***	0.979	0.960	0.966
**Distensibility**	-0.960	-0.981	-0.988
***PWV***	0.312	0.099	0.067
***ΔW***	-0.953	-0.958	-0.963

Correlation between four key calculated metrics (Pulse pressure *PP*, distensibility, pulse wave velocity *PWV*, and the change in stored elastic energy *ΔW*, each at the center of each of six key aortic segments; cf. Fig 6) and the associated prescribed circumferential material stiffness for a given age group.

**Table 6:** 

	Segment 1	Segment 2	Segment 3	Segment 4	Segment 5	Segment 6
***PP***	0.970	0.907	0.772	0.740	0.592	0.504
**Distensibility**	-0.983	-0.964	-0.946	-0.952	-0.944	-0.893
***PWV***	0.993	0.994	0.970	0.997	0.982	0.840
***ΔW***	-0.996	-0.993	-0.996	-0.999	-0.998	-0.939

Correlation between four key calculated metrics (Pulse pressure *PP*, distensibility, pulse wave velocity *PWV* and the change in stored elastic energy *ΔW*) and the prescribed circumferential material stiffness at the center of each of six key aortic segments (cf. Fig 6).

Computed values of aortic root-to-iliac and carotid-to-iliac *PWV* are reported in Table 7. These values are contrasted with measured values for *cf-PWV* for different age groups in a population with no risk factors [16,50]. Because methods for acquiring *PWV* in patients can vary in terms of calculation of transient time and path length, Boutouyrie and Vermeersch [16] standardized the transient time on the intersecting tangent algorithm and the path length on the direct distance. Because of the latter, they also used a scaling factor of 0.8 to convert the *PWV* obtained using direct distance to ‘real’ aorto-to-iliac *PWV*, since direct distance overestimates *PWV*. This scaling factor was reported by Weber et al. [51] based on comparisons of results inferred from both *cf-PWV* calculated with direct distance path length and aorto-to-iliac *PWV* calculated with an invasively measured real path length. In contrast, no scaling factor was used for the reported *cf-PWV* values measured by others [50]. These values, although measured between carotid-to-femoral, are compared directly to our carotid-to-iliac results since our geometrical models do not extend to the levels of the femoral arteries. Similar trends are observed over time between our results and the population data. Our results agree better, however, with population data in which aortic length was corrected.

**Table 7:** 

	Aortic Root-Iliac bifurcation *PWV*		Carotid-to-Iliac artery *PWV*	
Age	Computed	Reported [16]	Computed	Reported [50]
40 yo	6.9	6.9	9.7	7.75
60 yo	9.0	9.3	11.8	11.15
75 yo	11.0	10.9	14.4	13.8

Values of pulse wave velocity (*PWV*, in m/s) calculated over two segments, from the aortic root to the iliac bifurcation (left) and from the carotid artery to the iliac artery (right). Computations are compared with reported values by [16] and [50]. Values in [16] were originally measured as carotid-to-femoral, then scaled by 0.8 to represent aorto-iliac values, following Weber et al. [51]. Conversely, values in [50] correspond to carotid-to-femoral *PWV* with uncorrected path lengths. Our results show a better agreement with population data in which aortic length was corrected, as would be expected given our use of actual path lengths.

## Discussion

Although *cf-PWV* is considered by many as the gold standard clinical measure of arterial stiffness (e.g., [12,16]), it represents an average over a large portion of the arterial tree. Significant regional differences in aortic mechanics (geometry or properties) that could manifest with aging or disease and have detrimental effects on cardiac function can initially have limited impact on the numerical values of *cf-PWV*. The key question, therefore, is whether *cf-PWV* can detect early regional changes in aortic properties that could increase disease risk. Although this issue could be addressed via a lengthy clinical study, computational models enable time- and cost-efficient initial assessments. It was for such reasons that we developed a FSI model based on the best available, population-averaged data of arterial stiffness, *MAP*, *PWV*, and aortic radii and length found in literature.

Whereas the present 3D FSI study appears to be unique, Vardoulis et al. [52] used a 1D computational fluid dynamics model to assess potential effects of the location of a synthetic aortic graft on overall systemic hemodynamics. In particular, they contrasted effects of placing a graft in either the ascending or the descending thoracic aorta. Noting that such grafts introduce a highly localized change in aortic stiffness (modeled to be 3 to 5 times stiffer than the native aorta), it was found that an ascending graft augments the forward wave whereas a descending graft augments reverse flow (i.e., wave reflections). The former results in greater increases in central pulse pressure, thus they concluded that “patients who receive ascending aorta grafts are more prone to systolic hypertension.” Xiao et al. [35] performed a 3D computational study in which three different distributions of aortic stiffness and the corresponding hemodynamics were investigated on a fixed arterial model. Results demonstrated well-known trends of increased *PWV* and *cPP*, but reduced PP amplification, with increased levels of stiffness.

It should be noted that we focused on but three ages, ~40, 60, and 75 yo. The subject-specific 30 yo model was not analyzed due to the lack of data for wall properties of the ascending aorta at this age, where extensibility and distensibility are very important. There is clearly a need for more data focusing on this particular age range. The lack of simulations for ages ~ 50 to 55 was also intentional, for it is during this period that there tends to be dramatic changes in *cPP* [10], ascending aortic distensibility [50], and even brachial artery flow mediated dilation [14]. Hence, results could vary considerably depending on the specific values used for geometry and properties. Notwithstanding the need for a detailed analysis of the hemodynamics during this critical period of aging, using current values that are averaged over 50 to 55 years of age (cf. [21]) could be misleading and thus was avoided.

We examined spatial and temporal distributions of four different metrics of aortic stiffening: *PP*, *D*, *PWV* and *ΔW* relative to the distribution of circumferential material stiffness. As well known, there is a *PP* amplification along the aorta at the younger ages that diminishes over time due to an increase in *cPP* [49]. Higher changes in c*PP* between groups are expected if a younger age was considered, but as noted above this was not possible due to the lack of reliable data on ATA stiffness for a population younger than 40 yo.

Predicted values of *D* compared well with literature data. Redheuil et al. [50] reported values of distensibility for ATA of 3.1x10^-5^ Pa^-1^, 1.8x10^-5^ Pa^-1^, 1.2x10^-5^ Pa^-1^ and 1.0x10^-5^ Pa^-1^, for age groups 40–49, 50–59, 60–69, and >70 years old, respectively. Garcia-Herrera et al. [53] reported values of distensibility for the ATA of 2.0x0^-5^ Pa^-1^ and 0.98x10^-5^ Pa^-1^ for age groups 16–36 yo and 65–90, respectively. Koullias et al. [54] reported values of distensibility for the ATA of 1.87x10^-5^ Pa^-1^ in 64 years old patients. These values are in close agreement with our computations as shown in Fig 6, the exact numbers being: 3.27x10^-5^ Pa^-1^, 1.49x10^-5^ Pa^-1^ and 1.21x10^-5^ Pa^-1^ for 40, 60 and 75 years old age groups, respectively.

The distribution of *PWV* in Fig 6 reveals notable differences with age at segment 5 (renal arteries) and less remarkable variations at segments 4 (suprarenal abdominal aorta) and 6 (iliac artery) for the three ages. Given a certain age group, *PWV* is the only metric with low correlation coefficients relative to circumferential stiffness (Table 5), due to its dependency on non-normalized arterial geometry, which contrasted with the other metrics which are strictly related to the stiffness. Furthermore, *PWV* is often obtained clinically without measuring the actual path length between the two recorded waveforms. Boutouyrie and Vermeersch [16] used a 0.8 correction factor to relate the *cf-PWV* measured using direct distance to the ‘real’ aortic root-iliac bifurcation *PWV*. Indeed our aortic root-iliac bifurcation *PWV* compared well to their results (noting that the *TP* was tuned to match their *PWV* data). On the other hand, our results on carotid-to-iliac *PWV* show larger discrepancies with the values of *cf-PWV* reported by Redheuil et al. [50], who did not use correction factors for the aortic length. Use of the correct path-length is thus critical in computing *PWV*. Sugawara et al. [55] also investigated the importance of the 0.8 correction factor in the assessment of *PWV*.

The change in strain energy storage during the cardiac cycle was calculated using *ΔW* = 0.5(*P*_*sys*_*-P*_*dia*_)·(*A*_*sys*_*-A*_*dia*_)/*A*_*dia*_, which is an accurate estimate in all regions other than ATA. Unlike other aortic segments, the ATA experiences large deformations in the axial direction during the cardiac cycle, hence extensibility contributes to strain energy storage in the ATA. To properly account for this contribution to the ATA strain energy, our FSI computational model must be extended to include the large motion imposed by the heart on the aortic root and clinical data are needed to quantify actual changes in length over the cardiac cycle. We do not expect that this limitation has a significant impact on the computed values of *PWV*, *PP*, and distensibility in other parts of the aorta since these metrics are mainly determined by the arterial stiffness and the geometry of the vessel.

This work confirmed the importance of tethering exerted by perivascular tissue on the aorta [56]. Since reported values of external pressure exerted on the aortic wall are wanting (cf. [57]) we estimated them iteratively until our results satisfied typical values of tissue properties, *PP*, and *cf-PWV* for each age group. The pressure exerted by the perivascular tissue, fundamental to investigate *PWV*, was modeled via a simple traction boundary condition acting on the arterial wall. Modeling explicitly the interactions between aorta and surrounding organs would allow a thorough study of the contact stresses among the different tissues, but this was beyond the scope of this work. Dynamic data on wall motion would also allow one to identify regions with distinct levels of external support. Since all the models were aged virtually, we lack such data. Rather, we considered three (arbitrary and simple) regions of external tissue support with distinct values of external stiffness and damping.

It should be noted that as we adjusted the external tissue parameter *k*_*s*_, computed *PWV* and *PP* changed. Larger values of *k*_*s*_ result in larger *PWV* and *PP*. The sensitivity of these changes is small, however; it takes a 5-fold increase in *k*_*s*_ to increase *PP* by 50% and *PWV* by 250%. In the limit of very large external tissue support, the *PP* would approach that of a rigid vessel, and the *PWV* would become infinite. Tissue tethering affects motion in potentially all directions: radial, circumferential and axial directions. We focused on the role of tethering in the radial direction, which has a direct impact on *PWV*, distensibility, and other reported metrics. Time-resolved data would enable investigating the impact of tethering in the axial and circumferential directions.

Although the present model represents multiple important advances, there is yet a need for additional data to better inform it and a need for some computational improvements. Aging likely affects the peripheral vasculature and hence the associated outlet boundary conditions. Changes in *MAP* resulting from the increase in tortuosity with aging were not enough to reproduce the increase in *MAP*. Hence, we tuned the distal resistance *R*_*dist*_ of the Windkessel model at each age to match the *MAP*. This increase in peripheral resistance suggests that the distal vasculature becomes constricted with aging, which would be consistent with a myogenic-driven inward remodeling [58,59]. Having measurements of flow and pressure at the branches for each age group would allow us to estimate the values of the Windkessel parameters with more certainty. Given the evidence that the primary muscular arteries—such as brachial and radial and presumably mesenteric, renal, and celiac arteries—do not stiffen with aging [60], we assigned unchanged stiffness to these vessels for all age groups. Given the lack of data regarding the material properties of muscular arteries, we assigned stiffness values comparable to those in the aorta at the renal level at 75 yo. More information on these vessels is needed. Finally, from a computational perspective, our model must be expanded to account for the large displacements imposed by the heart on the aortic root.

In conclusion, advances in medical imaging and computational biomechanics now enable subject-specific models of large portions of the vasculature to be used to explore important interactions between the evolving wall mechanics and the hemodynamics. Such models reveal the need for more precise and complete regionally-specific longitudinal data, however, which promise to enable us to glean increased insight into the complex interactions between local mechanics / pathobiology and global hemodynamics / pathophysiology that relate central arterial stiffening to diverse diseases [61].

## Supporting information

S1 File vtk file (30yo.vtk) of the patient-specific aortic segmentation of the 30 yo volunteer. (VTK)

S2 File vtk file (40yo.vtk) of a virtually-aged aortic geometry with typical dimensions of a 40 yo individual. (VTK)

S3 File vtk file (60yo.vtk) of a virtually-aged aortic geometry with typical dimensions of a 60 yo individual. (VTK)

S4 File vtk file (75yo.vtk) of a virtually-aged aortic geometry with typical dimensions of a 75 yo individual. (VTK)
